# Stem cell treatments for female reproductive disorders: a comprehensive review

**DOI:** 10.1186/s13048-025-01750-y

**Published:** 2025-07-24

**Authors:** Ramya Nair, Prachi Agarwal, Mrunmayi Ashish Gadre, Kirthanshri S. Vasanthan, Raviraja N. Seetharam

**Affiliations:** 1 Manipal Centre for Biotherapeutics Research, Manipal Academy of Higher Education, Manipal, Karnataka 576104 India; 2 Manipal Centre for Biotherapeutics Research, Manipal Academy of Higher Education, Manipal, Karnataka 5776104 India

**Keywords:** Reproductive disorders, Women, Stem cell therapy, Mesenchymal stem cells, *In vitro* studies, Endometriosis, Exosomes

## Abstract

Stem cell research is advancing rapidly, offering substantial promise in reproductive medicine, particularly in addressing infertility and other reproductive disorders. Although recent advances have generated significant interest, the successful translation of stem cell treatments from preclinical research settings to clinical practice requires a comprehensive understanding of the underlying mechanisms and methodological approaches. This review assesses the current state of stem cell applications in the field of reproductive medicine, emphasizing current research and development, as well as the associated challenges. Adult stem cell-based interventions show considerable potential for treating reproductive tract disorders, mainly ovarian and endometrial regeneration. Despite these promising developments, the transition to widespread clinical implementation is hampered by several challenges, including its heavy reliance on preclinical animal data. The promise of stem cell therapy is considerable, however, validated mechanisms need to be developed that can fully harness their therapeutic capabilities in clinical settings. This review consolidates and evaluates the evidence regarding the therapeutic potential of various stem cell sources, emphasizing their benefits and drawbacks. Although stem zcell therapies have substantial potential for rejuvenating organ dysfunction, future research studies should focus on defining methodological enhancements, such as improving stem cell delivery methods and ensuring long-term safety, to overcome current limitations.

## Graphical Abstract

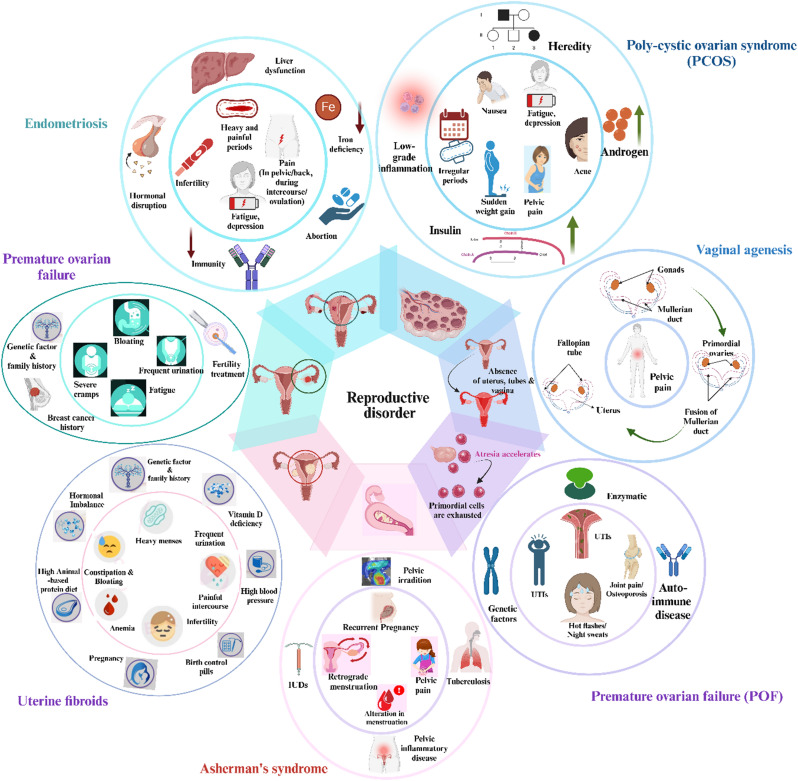

## Introduction

**Fig. 1:**
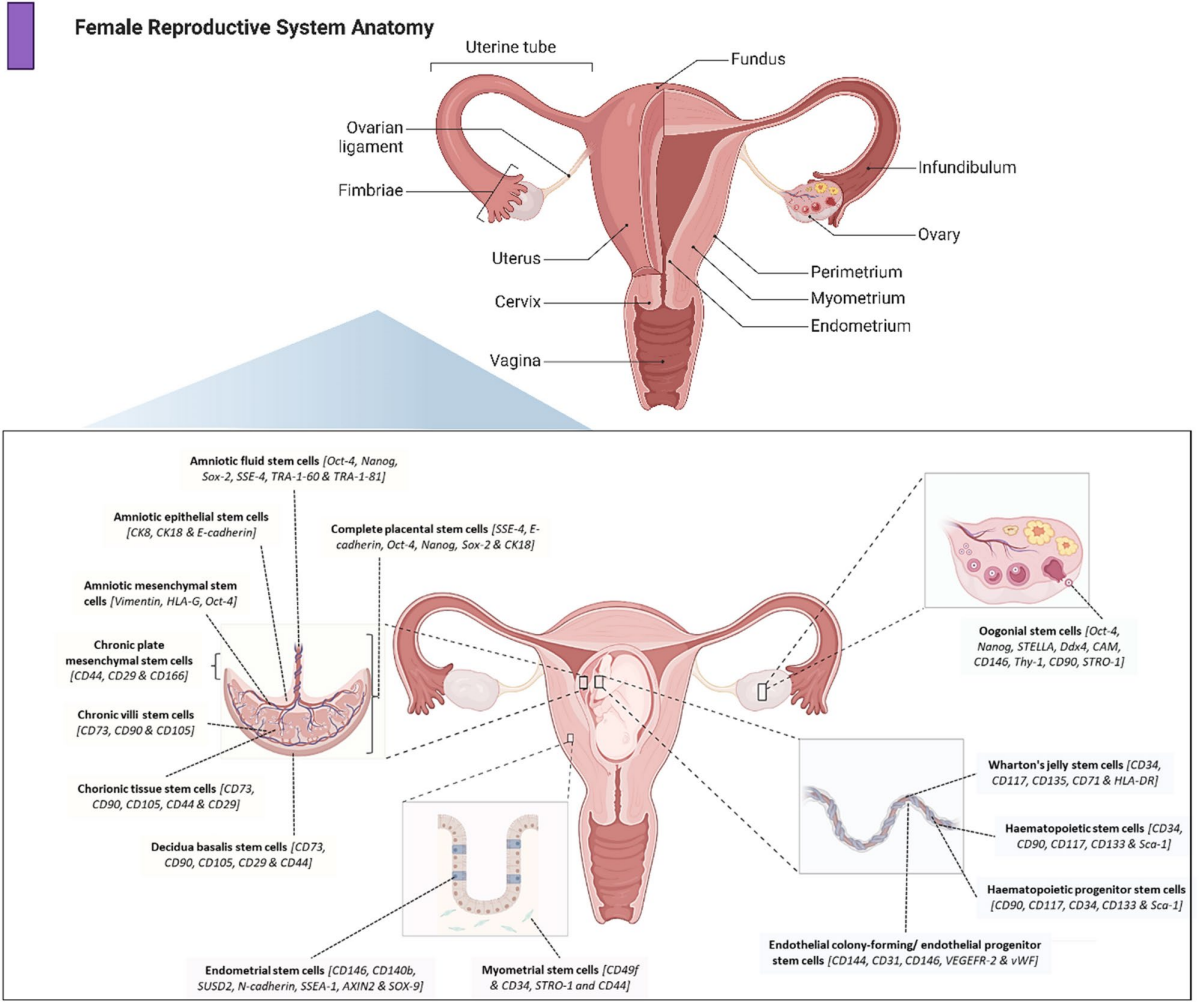
Representative image of the female reproductive system and the types of stem cells that can be isolated from these organs. Distinct stem cell populations can be derived from reproductive organs such as the endometrium, ovary, placenta, and umbilical cord, along with their representative cellular markers. Images were created using Biorender software

Female reproductive disorders affect millions globally, affecting both individual health and community well-being. These disorders contribute significantly to public health challenges, including infertility and complications related to maternal and perinatal health. The female reproductive system is comprised of a network of organs and regulatory pathways crucial to women’s physical and mental well-being, extending beyond reproductive health. Figure 1 shows a representative image of the female reproductive system and its constituent organs. Epidemiological studies indicate an increasing prevalence of reproductive disorders, especially in developing countries, which are influenced by environmental factors and lifestyle changes [1].

The female reproductive system performs various functional activities regulated by precise hormonal manipulation. Even a subtle disruption in hormonal release can lead to serious health complications that include not only direct reproductive challenges such as gestational disorders and pregnancy complications but also systemic health issues affecting the cardiovascular, skeletal, and metabolic systems [2, 3]. Studies have revealed a bidirectional relationship between reproductive health and systemic wellness through the correlation between ovarian hormone imbalances and the development of conditions, such as osteoporotic cardiovascular diseases and various organ-specific pathologies that affect the brain, kidneys, and liver [3, 4]. Dysfunction of neuroendocrine feedback mechanisms is often linked to these conditions [5].

The World Health Organization (W.H.O.) reported data concerning female reproductive disorders, with ovulatory dysfunction accounting for 25% of significant infertility cases. Other predominant cases reported included endometriosis (15%), pelvic adhesions (12%), tubal blockage (11%), and other tubal or uterine abnormalities (11%) [6]. Conventional treatments include lifestyle modifications, hormone replacement therapy (HRT), ovulation induction medications, and advanced assisted reproductive technology (ART). Although these methods have successfully improved the quality of life of patients, they are associated with an increased risks of pregnancy-related complications and cancer [7–10]. Given the limitations of current treatments, regenerative therapies, particularly stem cell-based approaches, have emerged as promising alternatives for restoring physiological function.

Regenerative therapy for the management of reproductive disorders is an alternative to existing treatment approaches [11]. Stem cell therapy restores functions through tissue repair and immune modulation rather than merely managing symptoms. Figure 1 illustrates the complex anatomy of the female reproductive system, underscoring the multifaceted targets of regenerative interventions discussed in this review. While several reviews have summarised the role of Mesenchymal stem cells (MSCs) in reproductive disorders, this review provides a broader, more integrated perspective by critically evaluating the full spectrum of stem cell sources from embryonic to induced pluripotent to reproductive-origin MSCs. This review further distinguishes itself by critically evaluating acellular derivatives (exosomes and secretome) and analysing India’s regulatory framework for stem cell therapies, a perspective absent in global reviews. This review also evaluates preclinical and clinical research outcomes, while dissecting the underlying mechanism of action to provide a comprehensive understanding of this emerging therapeutic approach. In addition, we discuss the future directions and necessary developments to advance this promising field.

## Stem cell therapy

The use of stem cells for therapeutic interventions signifies a transformative approach in the treatment of a range of disorders. Their ability to secrete paracrine factors that can promote tissue repair and homeostasis, activating distinctive molecular signatures and complex regulatory networks, adds to their clinical value. These characteristics are determined by their origin and differentiation characteristics. Stem cells are categorised based on their developmental potential and lineage specificity as shown in Table 1. The therapeutic abilities of embryonic stem cells (ESC), induced pluripotent stem cells (iPSCs) and MSCs in alleviating female reproductive disorders are detailed in the following subsections. For female reproductive disorders, ESCs and iPSCs enable tissue replacement through differentiation into reproductive cell lineages. MSCs, due to robust paracrine activity through their secretome, enriched with growth factors, cytokines, immunomodulatory factors (IL-10, TGF-β, PGE2), and angiogenic molecules (VEGF, bFGF, PDGF) activate multiple tissue repair pathways demonstrating most clinical significance [13]. Encouraging results from preclinical studies and early clinical trials for primary ovarian insufficiency and Asherman syndrome have underscored their significance. Despite encouraging developments, stem cell therapies face challenges that require resolution before clinical implementation, such as optimising delivery methods, assessing long-term effects, standardising protocols, and ensuring safety.

**Table 1:** Classification of stem cells based on the lineage commitment and differentiation abilities

Type	Developmental Potential	Key Regulators	References
Totipotent	All embryonic & extraembryonic cells	MERVL, DUX	[12]
Pluripotent	All three germ layers (iPSCs, ESC)	OCT4, SOX2, NANOG; SSEA3/4, TRA-1-60/81 markers; PI3K/AKT, WNT/β-catenin pathways	[12]
Multipotent	Lineage-restricted (Mesenchymal stem cells)	Tissue-specific transcription factors	[13, 14]
Unipotent	Single cell type (Oogonial, Spermatogonial)	Unidirectional differentiation signals	[12]

**Table 2:** Comparison of stem cell types used for treating female reproductive disorders

Feature	Embryonic Stem Cells (ESCs)	Induced Pluripotent Stem Cells (iPSCs)	Mesenchymal Stem Cells (MSCs)
Source	Inner cell mass of blastocyst	Somatic cells (e.g., fibroblasts) reprogrammed by OCT4, SOX2, KLF4, c-MYC	Adult tissues: bone marrow, adipose, endometrium, placenta, umbilical cord
Potency	Pluripotent (can form all three germ layers)	Pluripotent (can form all three germ layers)	Multipotent (differentiates into mesodermal lineages: bone, fat, cartilage; limited endoderm/ectoderm potential)
Key Markers	OCT4, SOX2, NANOG, SSEA-3/4, TRA-1-60/81	OCT4, SOX2, NANOG, SSEA-3/4, TRA-1-60/81	CD73, CD90, CD105 (positive); CD45, CD34, CD14, CD19, HLA-DR (negative)
Main Mechanism	Direct differentiation into somatic and germ cells, serves as tissue replacement	Direct differentiation into somatic and germ cells	Paracrine signaling (cytokines, growth factors, exosomes), immunomodulation, limited differentiation (endometrial and granulosa cells)
Signaling Pathways	PI3K/AKT, JAK/STAT3, WNT/β-catenin, SMAD, BMP, LIF	PI3K/AKT, WNT/β-catenin, SMAD, Activin A, BMP, DAZL, STRA8, DMC1	WNT/β-catenin, Notch, Hedgehog, VEGF/VEGFR2, MAPK/ERK, STAT3, FOXO3a, CXCR4/SDF-1
Reproductive Applications	Ovarian and endometrial regeneration, in vitro gametogenesis, delay of ovarian aging, restoration of fertility in animal models	Generation of oocyte-like cells, personalised fertility treatments, potential for autologous transplantation	Ovarian regeneration (POI), endometrial repair (Asherman syndrome), modulation of inflammation (endometriosis), angiogenesis promotion
Advantages	High plasticity, robust differentiation,	Avoids ethical issues, personalized therapy, scalable	Immunomodulatory, anti-inflammatory, low immunogenicity, easy isolation, fewer ethical concerns, well-tolerated in clinical studies
Limitations	Ethical/legal concerns, risk of teratoma, immune rejection, regulatory hurdles, limited clinical translation	Genomic instability, risk of teratoma, technical complexity, low germ cell yield	Donor variability, potential senescence, less robust for direct tissue replacement
Clinical Evidence	Preclinical rodent/primate studies	In vitro and animal studies; early clinical trials in other fields, not yet standard for reproductive disorders	Multiple preclinical and clinical studies for reproductive disorders, ongoing clinical trials
Key Challenges	Standardization of differentiation, control of epigenetic reprogramming, ethical oversight, tumorigenicity	Genomic/epigenetic instability, efficiency of differentiation, safety and standardization	Standardization of protocols, long-term safety and efficacy, donor-to-donor variability

### Classification of stem cells in therapeutic applications

Pluripotent stem cells, including both ESC and iPSCs, and adult stem cells, have demonstrated significant therapeutic potential in addressing various female reproductive disorders. The female reproductive system contains a dynamic population of stem cells essential for normal reproductive functions that play key roles in uterine regeneration and tissue remodeling (Fig. 1). The ovarian niche also harbors stem cells that replenish the pool of primordial germ cells. These stem cells, obtained from various endogenous reproductive sources, play a significant role in the treatment of reproductive disorders. This section discusses the immense potential of stem cells in in vitro gametogenesis and bioengineering as a therapeutic modality for personalized treatment of female reproductive disorders.

#### Embryonic stem cells

The pioneering work of Thomson et al. [15] opened new avenues in regenerative medicine by deriving human embryonic stem cells (hESCs) from the inner cell mass of the blastocyst. Subsequent studies led to the successful derivation of hESCs from embryos in the early preimplantation stage, such as the morula and 8-cell stages. These cells exhibit distinctive surface markers (SSEA-3/4 and TRA-1-60/81), expression of pluripotency transcription markers (OCT4, NANOG, and SOX2), and high telomerase activity, which helps to maintain chromosomal integrity during self-renewal. hESCs exhibit remarkable plasticity and the ability to differentiate into the three germ layers (endoderm, mesoderm, and ectoderm) while maintaining genetic stability through precise epigenetic regulation. However, social and ethical challenges have limited widespread adoption in research and clinical contexts.

**a) Preclinical studies**: Recent preclinical investigations have shown promising advancements in the field of gametogenesis and fertility restoration through stem cell differentiation. In murine models, initial proof of concept studies has successfully demonstrated in vitro gametogenesis, generating post-meiotic oocytes and spermatozoa through the precise temporal regulation of developmental signaling pathways in embryonic stem cells [16]. Hayashi et al. [16] described a two-step differentiation process in which mouse embryonic stem cells are converted into primordial germ cells and epiblast-like cells, facilitated by the application of various growth factors. Supplementation with growth factors such as bone morphogenetic proteins (BMP4, BMP8b), leukaemia inhibitory factor (LIF), Stem Cell Factor (SCF), and Epidermal Growth Factor (EGF) initiated the primordial germ cell activation pathway. BMP4 and BMP8b activate SMAD1/5/8 signaling, whereas LIF activates the STAT3 pathway. In addition, SCF and EGF enhance cell proliferation by activating the PI3K/AKT pathway. Supplementation with retinoic acid and BMP2 further induced meiosis in germ cells by upregulating STRA8 and DMC1 [17]. Germ cells were successfully generated from primate embryonic stem cells through a modified induction protocol [18]. This variation in the protocol accounts for the species-specific variation in developmental timings and signaling requirements, highlighting the necessity for protocol modifications that account for species-specific developmental cues. However, precise control of epigenetic reprogramming, chromosomal stability during differentiation, and complete meiotic progression in vitro requires further standardization of protocols.

In parallel, Shin et al., [19] explored the therapeutic potential of human ESC-mesenchymal progenitor cells (MPCs) in delaying ovarian aging. Their research demonstrated that intravenous injection of these cells (5 × 10^6^ cells) every month consecutively for four months resulted in pregnancy in a 10-month-old natural perimenopausal mouse model. Treatment increased primordial follicles, restored the estrous cycles, normalised estradiol levels, and reduced ovarian fibrosis by downregulating inflammation and fibrosis-related genes through myeloid-derived suppressor cell suppression. In primates, this strategy was further validated, with intraovarian injection of human embryoid body-derived cells extending reproductive lifespan in perimenopausal Cynomolgus monkeys, leading to successful pregnancies. Treatment decreased ovarian fibrosis and DNA damage while enhancing sex hormone secretion [20].

These studies indicate the potential of these stem cells in treating patients experiencing complete germ cell depletion and who require fertility restoration. Due to their differentiation abilities,

#### Induced pluripotent stem cells

The reprogramming of somatic cells to iPSCs by the ectopic expression of four key transcription factors, OCT4, SOX2, KLF4, and c-MYC, was possible through the revolutionary discovery of Professor Shinya Yamanaka [21]. Establishing a pluripotent transcription network involves extensive epigenetic remodeling, such as DNA demethylation, histone modifications, and chromatin restructuring. The characteristic features of these cells include a high nuclear-cytoplasmic ratio, increased telomerase activity, and expression of pluripotency markers such as SSEA-3/4 and TRA-1-60/81 [22]. iPSCs can differentiate into different cell types and address associated ethical concerns. The scalability and diversity of iPSCs significantly enhance research and therapeutic endeavors.

The initiation of Japan’s inaugural clinical trial employing iPSCs [21] for the treatment of macular degeneration was a landmark trial that illustrated the worldwide adoption of stem cell technology in therapeutic contexts. This success has prompted numerous clinical investigations, and more than thirty-three clinical trials are currently exploring the therapeutic potential of differentiated cells originating from pluripotent stem cells for various diseases.

**a) Preclinical studies**: A significant breakthrough in fertility research emerged with the successful differentiation of human induced pluripotent stem cells (iPSCs) into oocyte-like cells [23]. The direct differentiation protocol uses a temporal sequence of growth factors such as BMP4, WNT3A, and Activin A, recapitulating the developmental pathways by stage-specific activation of DAZL, STRA8, and DMC1 for follicle development. BMP4 binds to type I and II serine/threonine kinase receptors (BMPR1A/B and BMPR2 respectively), phosphorylates SMAD1/5/8 and translocate these proteins to form complexes with SMAD4. This results in the activation of PGC-specific genes BLIMP1 and PRDM14. WNT3A activates canonical β-catenin signaling by binding to Frizzled/LRP receptors, enabling nuclear translocation and further upregulating the genes required for PGC competence and TCF/LEF transcription factors. Activating these two pathways and Activin A signaling initiates the SMAD2/3 molecular pathway, thereby upregulating the cascade that leads to DAZL expression. DAZL is the master regulator of meiotic initiation and plays a vital role in stabilizing transcripts that encode synaptonemal complex proteins and in increasing STRA8 expression [24]. STRA8, a critical checkpoint in meiotic progression, promotes chromatin remodeling and activates premeiotic DNA replication machinery. The activation of DMC1 by STRA8 ensures accurate chromosomal segregation by facilitating the proper synapsis formation of homologous chromosomes. Activation of these pathway may represent potential mechanisms that enable the generation of oocyte-like cells characterized by the expression of essential molecular markers (GDF9 and ZP1-3) and functional attributes.

However, clinical translation of iPSCs faces critical challenges such as genomic instability, potential residual epigenetic memory, teratoma formation, low germ cell differentiation, and incomplete meiotic progression [25]. Further studies must be carried out with improved culture systems that incorporates physiological niche factors and advanced genetic screening strategies. iPSCs have the potential to provide personalized fertility treatments; however, rigorous safety assessments and standardized protocols are essential before their clinical use.

#### Mesenchymal stem cells

Mesenchymal stem cells (MSCs) that are multipotent stem cells originating from the mesodermal layer of adult tissues have a restricted self-renewal ability/ They are capable of activating key pathways, including the Wnt/β-catenin, Notch, and Hedgehog signaling pathways involved in cellular reprogramming and repair [26]. These cells can be isolated from adult tissues, including adipose tissue, bone marrow, peripheral blood, the placenta, and cord blood. The International Society for Cellular Therapy (ISCT) has established specific criteria for recognizing human MSCs (hMSCs). This includes the expression of cell-specific surface markers, such as CD73 and CD90, and the absence of negative markers, including of hematopoietic markers, such as CD45, CD34, CD14, CD11b, CD79α, CD19, and HLA-DR. In addition to cell surface markers, these markers should also possess the potential for in vitro differentiation into osteoblasts (RUNX2/OS2 pathway), adipocytes (PPARγ/C/EBPα axis), and chondroblasts (SOX9/TGF-β signaling) [27].

MSCs have emerged as a promising therapeutic modality for female reproductive disorders because of their dual mechanism of action, including paracrine signaling and tissue regeneration through differentiation. The paracrine activity of MSCs involves the secretion of multiple signaling molecules that activate multiple coordinated mechanisms. Proangiogenic factors such as vascular endothelial growth factor (VEGF) activate the VEGFR2/PI3K/AKT pathway and promote endothelial proliferation [28]. Immunomodulatory molecules such as IL10 suppress inflammatory responses via STAT3 signaling, TSG-6 inhibits the NF-κB pathway, and IL-1RA competitively blocks IL-1 receptor signaling. The growth factors present in these paracrine secretions, such as fibroblast growth factor (FGF), activate the MAPK/ERK pathway to promote cell survival, transforming growth factor-β (TGF-β) triggers SMAD2/3-dependent antiinflammatory responses, and stromal cell-derived factor-1 (SDF-1) mediates chemotaxis through CXCR4 signaling [28].

##### a) Preclinical studies

MSCs release growth factors that enhance ovarian function by promoting the maturation include GDF9, which activates the SMAD2/3 pathway in granulosa cells, and BMP15, which stimulates SMAD1/5/8 signaling [29, 30]. Cumulus cell expansion is achieved through the upregulation of PTGS2 (COX2), which enhances prostaglandin synthesis; an increase in HAS2 promotes hyaluronic acid production, and the expression of TNFAIP6 facilitates cumulus matrix formation [31, 32]. Additionally, these factors upregulate BCL2 via the PI3K/AKT pathway, inhibit the activation of the Caspase-3 cascade, and modulate the BAX/BCL2 ratio in granulosa cells [31, 32].

In addition to their ability to rejuvenate follicles and granulosa cells in the ovary, their role in alleviating endometrial disorders such as Asherman’s syndrome and endometriosis has also been reported. In a model of Asherman’s syndrome, Zhao et al. [33] demonstrated that MSCs derived from human menstrual blood promote the proliferation of endometrial cells through the activation of ERK1/2 and JNK signaling and the upregulation of cyclin D1 and cyclin E. A reduction in fibrosis was observed due to the activation of STAT3-dependent Smad/CTGF pathway modulation, decreased TGF-β1/Smad3 signaling, and regulation of matrix metalloproteinases. These paracrine mechanisms facilitate injury repair and reduce scar formation. Additionally, the ability of endometrial MSCs to differentiate into myofibroblasts by Activin A signaling upregulates the expression of connective tissue growth factor (CTGF activates the SMAD2/3 pathway), alpha-smooth muscle actin (ACTA2 activates serum response factor signaling), type I collagen (COL1A1 activates the TGFβ/SMAD pathway), and fibronectin (FN1 activates the TGFβ/SMAD pathway) [34]. This differentiation capacity and their ability to activate the STAT3-dependent Smad/CTGF pathway reduce endometrial fibrosis in rodent models. These findings highlight the therapeutic ability of MSCs in treating ovarian and endometrial dysfunction through their capacity for differentiation and paracrine signaling-mediated regulation of angiogenesis and inflammation.

MSCs can be obtained from reproductive and non-reproductive tissues. In the following sections, we briefly cover various tissue sources for MSCs that can be used as a therapeutic agents for female reproductive disorders.

##### Mesenchymal stem cells of non-reproductive origin

MSCs from non-reproductive origins have attracted considerable interest due to their varied origins and therapeutic potential. These cells, known for their ability for limited differentiation and secretion of growth factors and cytokines for tissue regeneration, can be isolated from multiple tissues, including bone marrow, and adipose tissue.

***i) Bone Marrow derived stem cells***.

Bone marrow derived MSCs (BM-MSCs) have emerged as a significant focus of research for their therapeutic potential ability to differentiate into osteogenic, chondrogenic, and adipogenic cell lineages, which is vital for tissue regeneration and repair. Moreover, they secrete various angiogeneic and anti-inflammatory factors at the site of tissue injury, contributing to their repair [35]. The major factors released by the BM-MSCs includes antifibrosis, antiinflammatory, antioxidative and antiapoptosis cytokines, which collectively improve ovarian folliculogenesis and restore ovarian functions [36]. Several studies will be discussed in subsequent sections that detail their effectiveness in restoring fertility by improving both ovarian and endometrial functions. Clinical trials utilising autologous BM-MSCs for ovarian function restoration in humans are discussed, highlighting their therapeutic potential. The positive markers for BM-MSCs include CD73, CD90, and CD105, whereas they lack CD34 and CD45 [37]. However, the invasive procedures required to harvest stem cells presents a limitation in their widespread usage.

***ii) Adipose derived stem cells.***

Adipose derived MSCs (AD-MSCs) are a promising cell source for regenerative medicine especially because of their ease of harvesting using minimally invasive procedures and their high proliferation and differentiation capabilities [38]. These multipotent stem cells can differentiate into multiple lineages and also secrete several growth factors similar to BM-MSCs at the site of tissue repair. The cell surface markers include CD90, CD29, CD59, whereas the negative markers include CD14, CD31, and CD19 [39]. Although there are currently no major clinical trials assessing the use of AD-MSCs for fertility restoration, several preclinical studies have examined their efficacy in ovarian and endometrial disorders that affect infertility.

##### Mesenchymal stem cells of reproductive origin

MSCs can be isolated from reproductive organs, including the uterus and ovaries, as provided in the schematic overview of the sources of reproductive tissues derived MSCs in Fig. 1. They offer distinct advantages over traditional sources such as the bone marrow. They are readily accessible during routine gynecological procedures, eliminating the need for additional invasive harvesting methods. These cells demonstrate robust proliferation capacity and possess immunomodulatory properties that are vital for maintaining reproductive function. They also exhibit enhanced expression of reproductive tissue-specific factors and epigenetic memory, favoring tissue-specific differentiation. Secretome composition may also include increased levels of reproductive tissue-specific growth factors and hormone-responsive signaling molecules [40]. This increases their therapeutic potential for reproductive disorders, as they may have improved macrophage modulation capability and enhanced survival in reproductive tissue environments. The following section comprehensively examines the unique biological properties of reproductive tissue-derived MSCs and their therapeutic applications in reproductive medicine.

***i) Uterine derived stem cells***.

The uterus exhibits dynamic properties and notable regenerative abilities, particularly during menstrual cycles and gestation. Stem cells in the uterus are located within the niches of both endometrium and myometrium are essential for various regeneration processes.

***ii) Endometrial stem cells and Menstrual blood derived MSCs***.

The endometrium consistently experiences concurrent degradation and repair processes throughout the menstrual cycle [41]. Endometrial stem cells in niches within the basalis layer of the endometrium, close to the blood vessels, are crucial for regenerating the functional layer during the menstrual cycle in response to hormonal variations [41, 42]. These cells demonstrate the ability to self-renew and possess the capacity to differentiate into various types of endometrial cell. The endometrium undergoes ~ 450 cycles of regeneration throughout the reproductive lifespan of females, and endometrial stem cells promote rapid re-epithelialization within 48 h [41, 43]. Endometrial MSCs (eMSCs) are characterised by their fibroblast-like morphology, and multilineage differentiated cells express specific markers, such as CD146, PDGFRβ (CD140b), and SUSD2 [44, 45]. The cells also exhibit clonogenic characteristics, as determined by their distinct markers (N-cadherin, SSEA-1, AXIN2, SOX-9, and ALDH1A1) [46]. MSCs isolated from menstrual blood (MenSCs) demonstrate consistent karyotypes over several passages and replicate approximately every 20 h at a notably faster rate than that of BM-MSCs [47]. eMSCs and MenSCs exhibit considerable therapeutic potential in endometriosis owing to their pronounced affinity for endometrial tissues. The characteristics and applications of these MSCs are listed in Table 3.

**Table 3:** Characteristics and therapeutic applications of mesenchymal stem cells in reproductive medicine

Cell Type	Source	Key Markers	Main Applications	Mechanisms
**Non-Reproductive Tissue-Derived MSCs**				
Bone Marrow MSCs (BM-MSCs)	Bone marrow stroma	CD73, CD90, CD105, CD44, CD29 (CD34⁻, CD45⁻, HLA-DR⁻)	POI, endometrial repair, ovarian dysfunction, male infertility	Trilineage differentiation, paracrine signaling, immunosuppression, angiogenesis
Adipose-Derived MSCs (AD-MSCs)	Subcutaneous/visceral adipose tissue	CD73, CD90, CD105, CD44, CD29, CD13 (CD34⁺, CD45⁻, CD31⁻)	Endometrial regeneration, ovarian rejuvenation, erectile dysfunction, vaginal atrophy	Enhanced proliferation, anti-apoptotic effects, neovascularization, tissue regeneration
**Reproductive Tissue-Derived MSCs**				
Endometrial MSCs (eMSCs)	Endometrium (basalis)	CD146, PDGFRβ, SUSD2, CD73, CD90	Asherman’s syndrome, endometriosis, thin endometrium	Paracrine repair, anti-fibrosis, angiogenesis, immunomodulation
Myometrial MSCs (MyoSCs)	Myometrium	CD49f, CD34, STRO-1, CD44, CD73	Uterine remodeling	Trilineage differentiation, tissue remodeling, wound healing
Menstrual MSCs (MenSCs)	Menstrual blood	SUSD2, CD146, PDGFRβ, CD73, CD90	Endometrial regeneration, Asherman’s syndrome, POI	Rapid proliferation, STAT3/Smad signaling, anti-inflammatory
Placental MSCs	Placenta (decidua, chorion, amnion), umbilical cord	CD73, CD90, CD105, CD29, CD44, CD166	POI, endometrial atrophy, ovarian dysfunction, pregnancy complications	Angiogenesis, immunomodulation, anti-inflammation, hormonal regulation
Ovarian Germline Stem Cells (OGSCs)	Ovarian cortex/tunica albuginea	Cytokeratin, embryonic/germ cell markers (OCT4, NANOG)	Infertility, ovarian aging (preclinical)	Oocyte-like cell differentiation, folliculogenesis support

eMSCs and MenSCs have shown promise in preclinical models for treating endometrial disorders such as Asherman’s syndrome and endometriosis. Their paracrine signaling supports endometrial repair, reduces fibrosis (via STAT3/Smad and TGF-β/Smad pathways), and promotes angiogenesis and antiinflammatory effects.

***iii) Myometrial Stem Cells***.

Myometrial Stem Cells (MyoSCs) support uterine plasticity and regenerative capacities during pregnancy. The uterus expands up to 1000-fold with a 20-fold increase in weight during pregnancy, which may reoccur over time, highlighting the importance of MyoSCs [48, 49]. The concept of MyoSCs was introduced by Ono et al. [50] due to the extensive transformations that occur in the uterine environment during embryo development. The longevity of the myometrium is linked to the minor population of cells in the G0 phase (characteristic of stem cells) [51]. The characteristic stem cell markers of MyoSCs are CD49f and CD34 [40] and STRO-1 and CD44 [34] and possess colony-forming ability and trilineage differentiation potential in vitro. However, owing to their limited availability, MyoSCs are not considered suitable for therapeutic uses.

***iv) Placental and Umbilical cord derived stem cells***.

The placenta is a vital temporary organ that facilitates fetal growth by providing essential nutrients and acting as an immune barrier throughout development. The umbilical cord consists of three components: the decidua basalis, chorion, and amnion. It is positioned centrally on the fetal aspect of the placental disc. The chorion and amnion are fetal layers, whereas the decidua is the modified maternal endometrium. In addition to tha characteristic placental cells, substantial amounts of stem cells are also present. MSCs can be isolated from the decidua basalis (DMSCs) and chorionic tissue (CMSCs) [52]. Amniotic epithelial cells are present in the epiblast layer of the amniotic membrane, and amniotic MSCs (AMSCs) are present in the hypoblast layer. MSCs can also be derived from amniotic fluid (AFMSCs) [53]. The MSCs derived from the umbilical cord and cord blood include Wharton’s jelly (WJMSCs), hematopoietic progenitor cells, hematopoietic stem cells (UCB–HPCs and UCB-HSCs), umbilical cord MSCs (UCB-MSCs), and endothelial colony-forming cells/endothelial progenitor cells (UCB-ECFCs/UCB-EPCs) [54] (Fig. 1). These noninvasive availability and unique properties of stem cells have are attracted increasing interest in clinical therapeutics and regenerative medicine. Placental stem cells possess the characteristics of both embryonic stem cells and MSCs, as they can differentiate into neurons in addition to trilineage cells.

Placental and umbilical cord-derived MSCs have demonstrated superior immunomodulatory properties and enhanced regenerative potential compared with BM-MSCs [55]. They are being explored for the treatment of ovarian insufficiency, endometrial atrophy, and other reproductive disorders because of their ability to promote angiogenesis, modulate inflammation, and support tissue repair [56].

***v) Ovarian Germline Stem Cells***.

The existence of ovarian germline stem cells (OGSCs), also referred to as oogonial stem cells, remains a subject of ongoing scientific debate. The long-held dogma of a finite ovarian reserve was challenged in 2004 after the first report of mitotically active ovarian germ cells by Johnson et al. [57] that year. In 2012, White et al. [58] documented the existence of mitotically active germ cells in women of reproductive age. The oogonial stem cell (OSC) pool first characterized by Bukovsky et al. in 2005 and located within the tunica albuginea lining [59]. Mesenchymal cells are positive for cytokeratin (CK) and differentiate through the mesenchymal-epithelial transition. SE cells form robust epithelial structures in the ovarian cortex and later migrate to the lower ovarian cortex, where they appear as small clusters. Differentiation of cells into granulosa, epithelial, neural, and mesenchymal types was effectively achieved under both estrogenic and non-estrogenic conditions. Successful differentiation into large oocyte-like cells, that exhibit characteristics commonly associated with secondary oocytes, was observed. The observed processes included germinal vesicle breakdown, polar body expulsion, and the expression of zona pellucida proteins [59]. Silvestris et al. demonstrated the potential to derive OSCs from human female ovaries, which can then be xenografted to facilitate their maturation into oocytes in vivo [60]. This finding aligns with previous findings that adult human ovaries contain bipotent progenitor mesenchymal cells that are capable of differentiating into granulosa and germ cells. This significant advancement facilitates novel strategies for egg preservation and treatment of female infertility, underscoring the potential advantages of utilizing fetal stem cells.

Nevertheless, the existence of OSC remains a subject of debate. An endogenous genetic approach demonstrated that germline cells in postnatal female mice do not undergo mitosis or participate in de novo folliculogenesis [61]. Despite significant interest from commercial entities in the potential application of presumed ovarian stem cells for infertility treatment, a consensus regarding their existence, origin, and functional roles remains elusive. Additional investigations are required to explore the issues related to OSCs.

Studies have indicated the clinical potential of these cells to restore ovarian functions in cancer survivors following chemotherapy. In a murine model, intraovarian injection of these germline cells (3 µl, ~ 2 × 10^4^ cells), resulted in the successful production of offspring, suggesting their potential as a novel therapeutic approach for treating ovarian failure and infertility. However, therapeutic effects are significantly influenced by several critical factors, such as the timing and dosage [62]. Although this study proves the ability of the germ cell line to restore fertility, the clinical translation of OGSC-based therapies faces limitations due to unresolved questions regarding their existence, origin, and functional significance. Additionally, contradictory findings [63] indicate that postnatal folliculogenesis may not occur in mammals, further emphasizing the necessity for continued investigation in this area.

vi) Very small embryonic-like stem cells.

Very small embryonic like stem cells (VSELs), which originate from bone marrow possess the potential to differentiate into three germ layers [64]. The presence of these cells is reported in the mouse uterine endometrium and myometrium apart from ovaries in mice. Despite not expressing MSC specific markers, they are negative for hematopoietic markers such as MHC-1 and HLADR. The markers specific to VSELs include stage-specific antigen A-1 (SSEA-1), Oct 4, Nanog, and Rif-1 telomerase protein. The expression of germ cell-specific markers Mvh, Stella, Fragilis, Nobox, and Hdac-6 is reported by few studies [65]. Although the functions of these are not clearly understood, it is believed that they migrate to the injury site and differentiate into various cell types found within the endometrium, including epithelial, glandular and myometrial cells, thus maintaining the uterine homeostasis [66]. Although research is ongoing regarding the application of VSELs in the treatment of erectile dysfunction, as well as neurological and cardiovascular disorders, further studies are required to explore their therapeutic potential in the treatment of endometrial disorders and infertility.

## Acellular stem cell therapy

Acellular stem cell therapy represents a cutting edge strategy in regenerative medicine effectively eliminating the requirement for direct stem cell transplantation. This therapy is based on the findings that their paracrine secretions mediate the therapeutic activity of MSCs, and that these molecules can initiate signaling pathways even in the absence of cells [67]. Compared with cellular therapy, the advantages of acellular therapy include reduced ethical concerns, immunogenic reactions, and the complexities of cell storage and transport. Several studies have established the efficiency of this therapy in treating a range of disorders within orthopedics, cardiology, and urology [67]. Furthermore, the utilization of acellular stem cell therapy in the reproductive field has been reported as a treatment for erectile dysfunction [68].

Exosomes are important components of the stem cell secretome, indicating their therapeutic value. These nanoscale vesicles (30–150 nm) are released from late endosomes and carry diverse molecules, including enzymes, heat shock proteins, microRNAs, DNA fragments, and proteins [69]. Exosomes can activate multiple signaling pathways in target cells, leading to cell proliferation and apoptosisby mitigating inflammation and decreasing apoptosis rates [70]. The therapeutic ability of exosomes originating from MSCs have been proven by various preclinical studies for liver disorders, gastric ailments, and other medical conditions, including brain injury [71].

Exosomes are identified by cell surface molecules such as tetraspanins and attach to target cells, whereas annexin and flotillin helps in the binding with target cells and release of components [72]. Other major cell surface proteins include major histocompatibility complex (MHC) class I and class II molecules and the intercellular adhesion molecules (IFMs) type 1, integrin, galectin, and collagen [73].Exosomes derived from oocytes are characterized by the presence of tetraspanin proteins (CD81 and CD9), and oocytes lacking these proteins resulted in polyspermy [74]. Immune modulation of UL16 binding protein 1–5 (ULBP1-5), MHCI, MHCII, and Fas ligand was observedthroughout the entire gestational period following the delivery of exosomes derived from the placenta [73].

### Therapeutic applications

Preclinical studies have reported that exosomes originating from the endometrium facilitates vascular development and maintains adequate blood circulation through the secretion of miRNA 21 and miRNA 126 [75]. The ability of exosomes as therapeutic agents to restore ovarian functions in a natural ovarian ageing mouse model has opened new avenues in acellular stem cell therapy. Exosomes derived from human umbilical cordMSCs (hUCMSCExos) decreased PTEN expression and inhibited apoptosis both in vivo and in vitro by the delivery of miR-21-5p [76]. The advancement of accessible treatments enhances availability and standardisation while presenting opportunities for cost reduction. Acellular stem cell therapy is increasingly being recognised as a vital element in future therapeutic strategies aimed at tissue repair and improving patient outcomes, driven by ongoing research advancements.

## Applications of stem cell therapy in female reproductive disorders

Female reproductive system-related disorders encompass a range of issues including ovulation disorders, irregularities in the uterus or cervix, damage to the fallopian tube, endometriosis, primary ovarian insufficiency, pelvic adhesions, and tumors, all of which lead to infertility. Stem cell therapy represents a novel regenerative technique aimed at alleviating female reproductive disorders and, offers a promising approach for treatment.

### Ovarian disorders

According to estimates from the World Health Organization (WHO), ovulatory abnormalities are responsible for 25% of cases of female infertility cases [77]. Anovulation and oligovulation are ovulatory disorders characterized by a lack of oocyte release each month, potentially leading to infertility. The World Health Organization has classified ovulatory disorders into four primary categories to improve their classification and management [78]. Among the various types of anovulation, normogonadotropic normoestrogenic anovulation is often linked to polycystic ovary syndrome (PCOS); hypogonadotropic hypogonadal anovulation, characterized by hypothalamic amenorrhea; hypergonadotropic hypoestrogenic anovulation, which encompasses conditions such as primary ovarian insufficiency (POI), Turner syndrome, and hyperprolactinemic anovulation, usually seen in cases of pituitary adenoma. Research on stem cell transplantation as a potential treatment for ovarian dysfunction and infertility is currently gaining momentum. Research has relied on MSCs derived from different sources, particularly bone marrow and umbilical cord, to increase ovarian angiogenesis and improve ovarian function.

#### Primary ovarian insufficiency

Primary ovarian insufficiency (POI) is a condition that leads to decreased ovarian function and irregular menstrual cycles in women under the age of 40 due to diminished ovarian reserve. The European Society of Human Reproduction and Embryology (ESHRE) describes POI as the condition characterized by the absence or irregularity of menstrual periods for four consecutive months, accompanied by elevated follicle-stimulating hormone (FSH) levels ≥ 25 IU/L [79]. Hypoestrogenism, follicle depletion, and menstrual irregularities lead to infertility in patients with POI, thereby, affecting their quality of life. POI can result from multiple factors, such as immune system disruptions, infections, genetic anomalies, and environmental factors, including chemotherapy. Autoimmune disorders account for 30% of patients. The prevalence of this condition is observed in 1 out of every 10,000 individuals within the 25 age group, 1 in 1,000 for those aged 30, and 1 out of 100 for individuals aged 40 years [80].

**i) Preclinical Evidence**

**Fig. 2:**
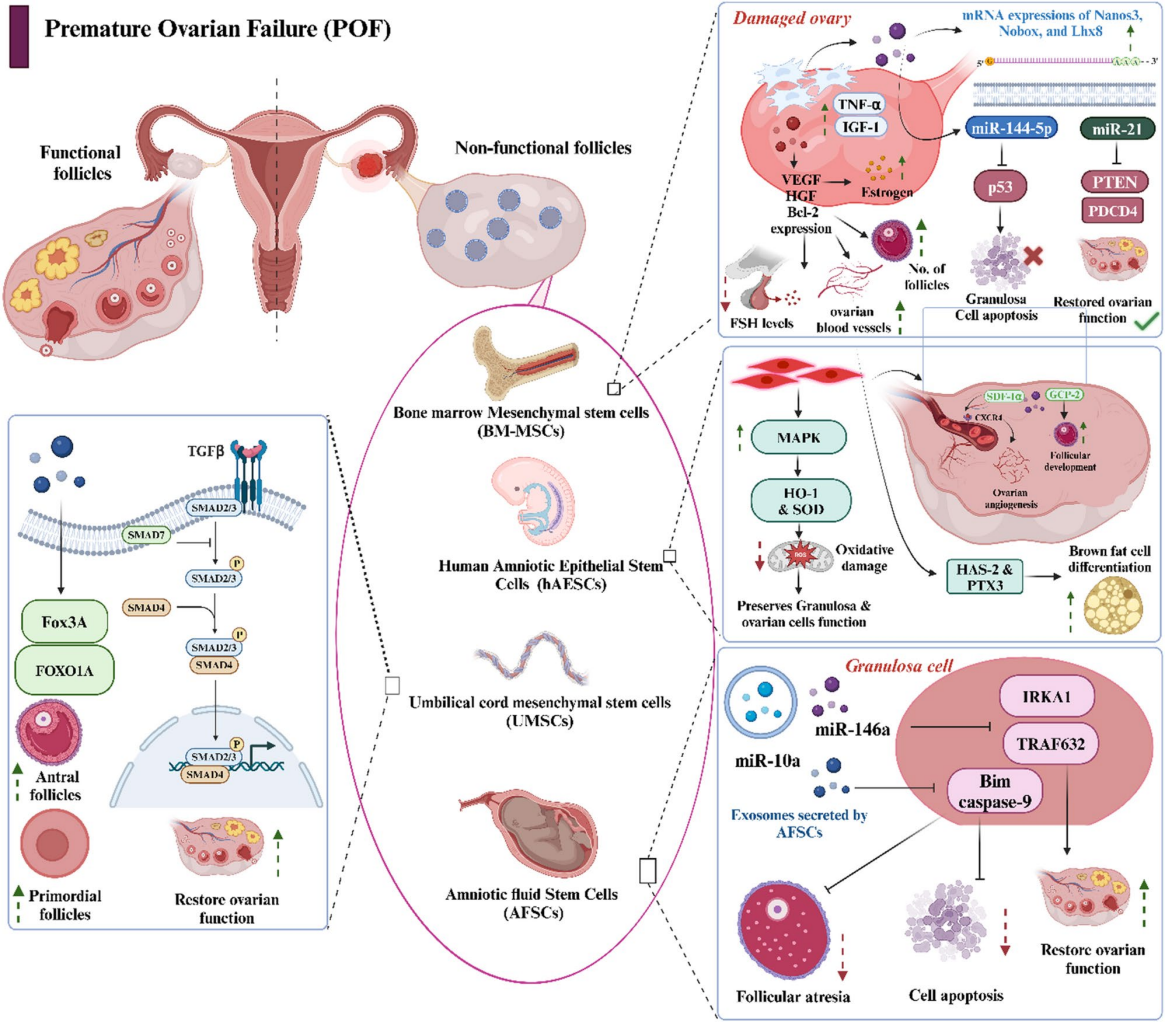
Impact of mesenchymal stem cells on primary ovarian insufficiency. The image illustrates the mechanism and potential treatments using mesenchymal stem cells and related molecular pathways involved in ovarian function. POF is characterized by nonfunctional follicles, which can be reversed using MSCs derived from bone marrow cells, human amniotic epithelial cells, the umbilical cord, and amniotic fluid. Key molecular pathways, including MAPK and TGF beta, are modulated by these MSCs, enhancing the proliferation of granulosa cells. Exosomes containing microRNAs (miR-146a and miR-10a) have been shown to further improve ovarian function. Images were created using Biorender software

The utilization of stem cells isolated from various sources, such as placental tissue, amniotic fluid, bone marrow, umbilical cord and adipose tissue, has demonstrated therapeutic potential animal models of POI (Fig. 2). Each source has demonstrated distinct therapeutic profiles by activating several interconnected mechanisms rather than direct cellular replacement, with paracrine signaling. The infusion of human amniotic epithelial stem cells (hAESCs, at 1–2 × 10^6^ cells/kg body weight) in the POI-induced murine model activated an antiinflammatory cascade, resulting in enhanced granulosa cell proliferation while diminishing germ cell apoptosis and mitochondrial dysfunction, reducing follicular atresia and promoting oocyte maturation and release. A decrease in the level of TNF-α, IL-1β and IL-6, alongside increased expression of the antiinflammatory mediators IL-10 and TGF-β was also observed in this study [78]. Transcriptomic analysis revealed the activation of the AKT and ERK signaling pathways, which are essential for the proliferation of granulosa cells and follicular activation [81]. Reduction in ovarian fibrosis as well as hormonal regulation was reported after intraovarian administration of human UC-MSCs (1 × 10^6^ cells/kg bw; i.v.) by decreased AMPK phosphorylation leading to the modulation of the AMPK/NR4A1 signaling pathways [82]. Administration of MSCs derived from bone marrow, placenta, umbilical cord, and adipose tissue [83–87] reported increasing VEGF levels, resulting in reduced FSH levels and increased estrogen levels. Similarly, MSCs sourced from the human chorionic plate and menstrual blood also restored ovarian function and hormonal regulation in murine POI models [88–92]. An elevation in the expression levels of the vascular endothelial growth factor-A gene and a corresponding increase in the density of blood vessels within ovarian tissues were noted following the administration of ADMSCs [93]. An alternative proposed mechanism for these paracrine factors suggests an elevated level of miR-21, down-regulating phosphatase and tensin homolog, and programmed cell death protein 4, thus reducing granulosa cell apoptosis in POF rat models [94].

Preclinical studies also investigated acellular therapy to elucidate the impact of paracrine secretions and exosomes as standalone therapeutic interventions for POI. Exosomes derived from human AD-MSCs were also reported to have similar effects, such as enhanced ovulation, granulosa cell proliferation, and corpus luteum formation after intraovarian administration in POI model mice (1 × 10^6^ cells/mL). These studies demonstrated effective downregulation of the Fas/Fas ligand (Fas/FasL) pathway and reduced the expression of caspase-8 and caspase-3 with a parallel elevation in SMAD5 expression [95]. However, long-term studies that can confirm the tumorigenicity and biodistribution have not been reported.

The ability of hADMSCs enhances the differentiation of peripheral blood mononuclear cells (PBMCs) from individuals with POI into regulatory T cells (Tregs) in the presence of 17β-estradiol indicates potential therapeutic applications in fields of immunotherapy and regenerative medicine [96]. The integration of molecular pathways revealed synergistic interactions between AKT/ERK activation and AMPK/NR4A1 signaling, promoting immediate cellular survival and long-term metabolic adaptation.

**ii) Clinical evidence**

The introduction of MSC cell therapy in patients was first introduced by Edessy et al. [97], where after autologous BM-MSCs were injected directly into the ovaries of 10 high-risk POI patients (Table 4). The patients reported successful resumption of menstruation after 3 months of infusion, and one patient (10%) resulted in the live birth of a healthy baby. In 2022, Naimat et al., [98] also reported the successful livebirth after the intraovarian injection of BM-MSC based on O-Cell^®^ Protocol of StemGenn Therapeutics in a 32 yeas idiopathic female. However, in this case, the protocol was performed twice (6-month intervals). With this success, they further injected 10 patients diagnosed with POF, POI or Low Ovarian.

**Table 4:** Table reporting registered clinical trials focused on using stem cell therapies for various reproductive disorders

No	ClinicalTrials.gov ID	Name	Status	Study Plan	Location
1	NCT05703308	Menstrual Blood Stem Cells in Poor Ovarian Responders	Completed	Interventional, Phase 3, MenSCs from patients were injected intravaginally at a density of 20 × 10^6^ cells/ml. Primary Outcome measured- Sponatneous pregnancy after 3 months of injection	Avicenna Research Institute, Tehran, Iran
2	NCT03069209	Autologous Bone Marrow-Derived Stem Cell Transplantation in Patients With Premature Ovarian Failure (POF)	UNKNOWN STATUS	Intraovarian transplantation of autologous purified bone marrow-derived stem cells and mesenchymal stem cells. Primary outcome: Return of menses in a woman with previous ameneorrhea for at least 6 months before recruitment.	Stem Cells Arabia, Jordan
3	NCT05495711	hUC Mesenchymal Stem Cells (19#iSCLife^®^-UT) Therapy for Patients With Thin Endometrial Infertility	RECRUITING	Biological: human umbilical cord mesenchymal stem cell complex collagen intrauterine injection with human umbilical cord mesenchymal stem cell; total 1 time injection with 1*10^7^ cells. Primary outcome: Pregnancy outcome	Sclnow Biotechnology Co., Ltd. Changsha, Hunan, China
4	NCT02151890	Pregnancy After Stem Cell Transplantation in Premature Ovarian Failure (POF)	COMPLETED	Biological: Stem Cell injection of stem cell sample in the ovaries of POF patients, Primary outcome: PregnancyHormonal analysis Cairo, Egypt	Edessy Mahmoud, Al-Azhar University
5	NCT02043743	Autologous Stem Cells Transplantation in Patients With Idiopathic and Drug Induced Premature Ovarian Failure	UNKNOWN STATUS	Biological: Biological: Stem Cells 3–5 million Bone marrow MSCs injected into ovarian tissue. Primary outcome: serum FSH, estrogen, AMH levels	El-Rayadh Fertility Centre, Giza, Egypt
6	NCT02372474	“It is a Real” The First Baby Of Autologous Stem Cell Therapy in Premature Ovarian Failure	COMPLETED	Biological: Stem cell laparoscopic injection of stem cell sample in the ovaries Outcome: Number of cases that go through menstruation, pregnancy and labour after stem cell transplantation in POF.	Edessy Mahmoud, Al-Azhar University
7	NCT04432467	Fertility Restoration Using Autologous Mesenchymal Stem Cells	COMPLETED	Biological: Autologous adipose tissue-derived mesenchymal stem cells. Patients with impending caesarean section or with chronic inflammation in the mucosa of the uterus and fallopian tubes receiving standard treatment and mesenchymal stem cells Outcome: Number of cured patients	Institute of Biophysics and Cell Engineering of National Academy of Sciences of Belarus, Minsk, Belarus Remove chrons disease
8	NCT06132542	Patients with premature ovarian insufficiency were transplanted with autologous adipose-mesenchymal derived stem cells for improving clinical hormone levels	NOT YET RECRUITING	Biological: Autologous adipose tissue-derived mesenchymal stem cells. The fat derived stem cell transplanted to either of the ovary. Outcom: Achievement of pregnancy, resumption of menses and improved clinical hormone levels.	Batsuren Choijamts, Mongolian National University of Medical Sciences
9	NCT02062931	Autologous Mesenchymal Stem Cells Transplantation In Women With Premature Ovarian Failure	UNKNOWN STATUS	Biological: Stem Cell Preparation and Injection Stem cells from platelets rich plasma (PRP) will be injected into ovarian tissues and ligaments. Stem Cell Dose: 3–5 Million Autologous MSCs. Outcome: Serum Hormonal level of FSH, estrogen, and AMH level.	Cairo, Egypt
10	NCT02603744	Autologous Adipose Derived Mesenchymal Stromal Cells Transplantation in Women With Premature Ovarian Failure (POF)	UNKNOWN STATUS	Biological: Intraovarian injection of Adipose derived Stromal cells (ADSCs) to ovary, 5–15 million MSC. Patient Outcome: Evaluation the ovary abcess or any other adverse events.	Royan Institute, Tehran, Iran, Islamic Republic of
11	NCT03592849	The efficacy and safety of collagen scaffold comprising of umbilical cord derived Mesenchymal Stem cells in infertile women with thin endometrium or having endometrial scarring	COMPLETED	Biological: UC-MSCs therapy Procedure: Women who were diagnosed infertile and having thin endometrium or endometrial scarring were transplanted with collagen scaffold loaded with UC-MSCs inside uterine cavity. Outcome: Endometrial thickness and Pregnancy	Nanjing Drum Tower Hospital of Nanjing University Medical School
12	NCT03877471	Transplantation of Mesenchymal Stem Cells (MSCs) into women having Primary Ovarian Insufficiency	UNKNOWN STATUS	Biological: Mesenchymal Stem Cells Procedure: The MSC-like cells were injection into each ovary through transvaginal ultrasound at three different injection locations. Injections were consisting of 2, 5 and 10 million in 100uL suspension on each ovary of the patient. Outcome: Patients will be looked for growing follicles which is stimulated by exogenous hormone which will be followed by retrieval of oocytes and in vitro fertilization (IVF).	Hongmei Wang, Chinese Academy of Sciences
13	NCT02144987	Treatment of Asherman’s Syndrome and Endometrial Atrophy with the application of Bone Marrow Stem Cell	COMPLETED	Biological: Bone Marrow Stem Cell (BMSC) was mobilized in peripheral blood. Procedure: The cells were induced by granulocyte-CSF (G-CSF) 5 mcg/kg for 4 days every 12 h. The transplantation of CD133 + cells was performed by intra-arterial catheterization into uterine spiral arterioles. Outcome: Increase in the live-birth rate, and higher implantation rate	Instituto Valenciano de Infertilidad, IVI VALENCIA, Valencia, Spain
14	NCT03166189	Treatment of women with repeated failure of IVF with increase in atrophic endometrium using the Autologous Bone Marrow-derived Mesenchymal Stem Cells	COMPLETED	Biological: bone marrow-derived MSC along with hormone replacement treatment Procedure: The treatment has endometrial injections that contained the cells. Along with this there was treatment of hormone replacement. The patients were observed for the combination of endometrial thickness and immunohistochemistry of endometrium biopsy after third cycle of treatment. Outcome: increase in atrophic endometrium of patients with repeated failure of IVF	Valeria Muller, D.O. Ott Research Institute of Obstetrics, Gynecology, and Reproductology
15	NCT00429494	Treatment by Hematopoietic Stem Cell Transplantation for ovarian function using GnRH Analogue	COMPLETED	Biological: Hematopoietic Stem Cell Transplantation Procedure: 22.5 mg of Leuprolide Acetate by injection intramuscular before and 3 months post transplantation. Transplantation involves administration of hematopoietic stem cell transplantation Outcome: Patients had improved ovarian function	M.D. Anderson Cancer Center
16	NCT02696889	Treatment of Patients with POF, POI or Low Ovarian Reserve with Bone marrow derived stem cell to improve hormonal levels	COMPLETED	Biological: Bone marrow derived stem cell fraction Procedure: Rejuvenation of Premature Ovarian Failure with Stem Cells Protocol was employed. Patients with POF, POI or Low Ovarian Reserve were injected with Bone marrow derived stem cell fraction into the right ovary. Outcome: Patients after treatment had improved hormonal levels with reduction of 50% in FSH values and increase in AMH and Estradiol levels (30%)	Ayman Al-Hendy, University of Illinois (Chicago)
17	NCT05138367	Transplantation of UCA-PSC and WJ-MSC in patients having POF	COMPLETED	Biological: UCA-PSC and WJ-MSC Procedure: Each ovary was injected with UCA-PSC and WJ-MSC (2 × 10^7cells, 1 × 10^7 /400 µL for unilateral ovarian injection) Outcome:	Li-jun Ding, Nanjing University
18	NCT03033277	Patients with Primary Ovarian Insufficiency (POI) was transplanted with Human Umbilical Cord Mesenchymal Stem Cells (HUC-MSCs)	UNKNOWN STATUS	Biological: Human Umbilical Cord Mesenchymal Stem Cells Procedure: Using the ultrasonic guidance, HUC-MSCs were injected via vagina Intraovarian Outcome: Patients had increase in the number of mature follicles	Hongmei Wang, Chinese Academy of Sciences
19	NCT02644447	Patients with POF were transplanted with HUC-MSCs With Injectable Collagen Scaffold	COMPLETED	Biological: HUC-MSCs Procedure: Bilateral ovaries injection was given with allogeneic HUC-MSCs (10 million). This transplantation was performed using Collagen Scaffold. Outcome:	Jianwu Dai, Chinese Academy of Sciences
20	NCT05308342	Treatment of Premature Ovarian Insufficiency using Human Umbilical Cord Mesenchymal Stem Cells	RECRUITING	Biological: Human Umbilical Cord Mesenchymal Stem Cells Procedure: Patients were transplanted with Human Umbilical Cord Mesenchymal Stem Cells into ovaries (10 × 106 cells, 5 × 106 for unilateral ovarian injection) guided by TVUS. Outcome: Higher activity rate of Follicular development rate and hormonal examination was observed.	Li-jun Ding, Nanjing University
21	NCT05279768	Patients With Insulin Resistance and Polycystic Ovary Syndrome were given by Infertility Therapy using Stem Cells and Secretome	RECRUITING	Biological: Stem Cells and Secretome Procedure: patients were given with tablets and nasal drop having UC-MSCs and Secretome once in 30 days (0.3 million kg/bb UC-MSCs and nasal drop 0.5 ml/day Secretome) Outcome: There was an increase in cytokine/adipokine/hormone profile. It was done by sampling of the patient’s blood serum during the follicular phase on day 10–12.	PT. Prodia Stem Cell Indonesia
22	NCT03535480	Patients were administered Autologous Bone Marrow Stem Cell to Restore Ovarian Function in Premature Ovarian Failure (ASCOT-2)	UNKNOWN STATUS	Biological: Autologous Bone Marrow Stem Cell Procedure: Patients were transplanted Autologous Bone Marrow Stem Cell and post this Granulocyte colony stimulating factor was administered subcutaneously during five days for stem cell mobilization via infusion in ovarian artery Outcome: There was an increase in Antral follicle count (AFC) in the patients	Antonio Pellicer, Instituto de Investigacion Sanitaria La Fe

Reserve as per ESHRE criteria and significant improvement in antral follicle count, anti-Müllerian hormone (AMH) levels, as well as fibroblast growth factor-2 (FGF-2) and thrombospondin (THSP-1) were reported in these patients. Successful pregnancies were reported in 6 patients (5 embryo transfer and 1 by natural conception) post protocol. The euploidy of the embryo was also normal when analysed by preimplantation genetic screening in these patients. In a large cohort study by Zafardoust et al., [99], intraovarian injection of MenSCs into the ovaries of 90 patients had no safety concerns raised. This treatment led to the increased release of mature oocytes, and a 22.5% pregnancy rate was reported (18 out of 80 individuals). No harmful side effects were observed after 3 months of follow-up. An increase in AMH levels and antral follicle count was also reported in these patients.

Currently, around 15 registered clinical trials (Table 4) are registered for MSC therapy for POI, and the data revealed significant heterogeneity in study design, patient populations, and therapeutic protocols. Sample sizes ranged from 2 to 90 patients, with 60% of studies reporting unknown or incomplete status, highlighting challenges in clinical trial completion and follow-up. Cell doses varied substantially from 2 × 10⁶ to 20 × 10⁶ cells per treatment, delivered through either laparoscopic or transvaginal ultrasound-guided injection approaches to the ovary. Primary analysis of hormonal restoration has been reported in these cases even after 1 year of treatment [100]. The secondary outcome, recovery of menstrual function, was also documented across multiple studies, with patients experiencing resumed menstruation within 3–7 months post treatment. However, long-term safety data remain limited, with most studies providing follow-up periods of 6–12 months.

#### Polycystic ovary syndrome

**Fig. 3:**
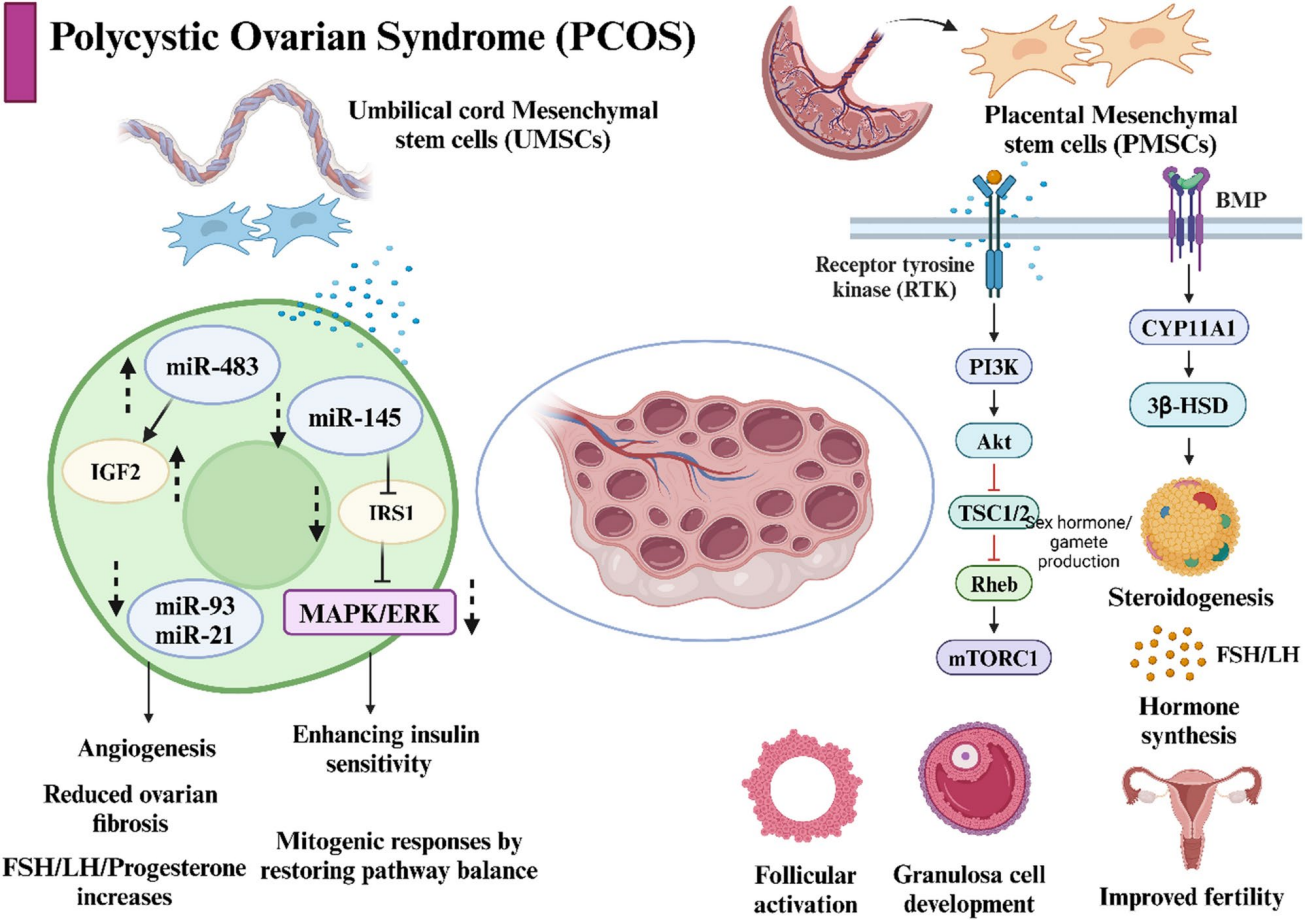
Effect of mesenchymal stem cells in alleviating polycystic ovarian syndrome. The MSCs treatment improved angiogenesis, insulin sensitivity, and hormone imbalance in PCOS conditions. Activation of PI3K/Akt and MAPK/ERK pathways by MSC secretome and microRNAs restored ovarian function. Images generated using Biorender software

Polycystic ovary syndrome (PCOS) is a widespread endocrine disorder that is associated with obesity and infertility. It affects 15–20% of women, especially in the reproductive window, with variations influenced by demographic factors and the criteria used for diagnosis. The prevalence of PCOS in India is approximately 11.34% [101]. The pathogenesis of this complex disease involves intrinsic interactions among genetic, metabolic, and environmental factors. This medical condition is characterized by anovulation, irregular menstrual cycles, elevated androgen levels, and the presence of multiple cysts in the ovaries. It is also related to metabolic conditions, including insulin resistance, diabetes, an elevated risk of cardiovascular disease, and mental health challenges. The management of PCOS requires a holistic approach that addresses both the reproductive and metabolic aspects of the condition in alignment with conventional symptom-based treatments. The primary etiologies of PCOS include inflammation, hyperandrogenism, and insulin resistance [102]. Notably, MSCs possess significant antiinflammatory properties, as illustrated in Fig. 3.

**i) Preclinical Evidence**

Intravenous injection of placental MSCs in PCOS induced Wistar rat models resulted in improved growth of preantral follicles with a simultaneous decrease in the incidence of cystic follicles [103]. A holistic improvement in PCOS conditions with reduction in elevated levels of testosterone and LH, increased FSH levels, increased insulin sensitivity, reduced fasting blood glucose levels, and optimised lipid metabolism and liver function was observed in these rat models. Research has demonstrated that PDMSCs influence their environment via paracrine signaling mechanisms, which activate various pathways, including the PI3K/AKT, ERK, and BMP signaling pathways essential for follicular growth, the functionality of granulosa cells, and the regulation of glucose metabolism [104].

Several other studies have also demonstrated enhancements in endocrine function, oocyte quality, and folliculogenesis associated with the antiinflammatory, antiapoptotic, and antioxidative activities of BM and UC MSCs following injection in the context of PCOS [105, 106]. The observed effect was facilitated by a reduction in the synthesis of proinflammatory cytokines (IL1β and TNF α), along with a decrease in gene expression implicated in the development of fibrosis, such as connective tissue growth factor. Concurrently, anti-inflammatory substances, notably IL-10, are elevated within local ovarian and uterine tissues [107, 108].BM-hMSCs have been found to suppress inflammation associated with PCOS by secreting IL-10 [109, 110]. In vitro studies utilising H295R cells in conjunction with theca cells obtained from PCOS-affected individuals revealed that the secretome of BM-hMSCs significantly hindered steroid generation, inflammatory reactions, and androgen synthesis [111]. In another study, apart from restoration of hormonal functions and follicular development, a decrease in the level of collagen and blood volume was reported in PCOS induced Wister rat models after injection of human MenSCs (1 × 10^6^ cells in 40 µL) [112].

Exosomal microRNAs present opportunities for acellular stem cell therapy, as demonstrated by the research conducted by Tamaddon et al. [113]. The secretome of hMSCs contains various factors that are capable of inhibiting androgen production in theca cells. This inhibition occurs through the downregulation of steroidogenesis related proteins, specifically the gene expression of aromatase synthase, CYP11A1, and CYP17A1, which are typically elevated in PCOS patients [106, 114]. The infusion of MSCs/exosomes decreased the concentration of proinflammatory cytokines, particularly IL-6 and TNF-α, and a decrease in malondialdehyde (MDA) levels, thereby increasing antioxidant capacity. Many studies have demonstrated a decline in proinflammatory cytokines, particularly IFN-γ, TNF-α, IL-6, and IL-17, while concurrently elevating the expression of anti-inflammatory cytokines, such as IL-4 and IL-10 [115–117]. A reduction in the inflammatory response is expected to decrease the symptoms associated with PCOS [118]. These studies emphasise stem cell therapy as a holistic treatment approach for PCOS conditions, and the long-term effects of these therapies are necessary for clinical translation.

**ii) Clinical Evidence**

Currently, one clinical trial is in progress, in which patients with insulin-resistant PCOS are administered as tablets and nasal drops containing UC-MSCs and secretome once every 30 days. This research is still under study, and no data are currently available. In another case study, as reported in a 28-year-old PCOS patient, UCMSC was administered underneath the skin of the scalp (130 million). Multiple subdermal injections were provided, totalling 20 million AlloRx stem cells. After 4 months of follow-up, apart from hair thickening, the patient reported normalisation of the menstrual period. After 10 months of follow-up, the ovarian cysts were completely absent after ultrasound, and after 18 months of follow-up, the patient did not report any adverse events [119].

These clinical data provide new hope for PCOS patients because a holistic treatment approach can be provided using stem cell therapy.

### Uterine disorders

#### Endometriosis

Endometriosis is defined as atypical growth of tissue outside the uterine cavity, primarily in the pelvic region. However, it may spread to other parts of the abdominal cavity. This condition affects a total of 10–15% of women of reproductive age, and 40–50% potentially experience infertility [120]. Endometriosis is categorized into four stages: minimal (stage I) to severe (stage IV). Endometriosis is a multifaceted pathological condition characterized by inflammatory processes, angiogenesis induction, and inherent resistance to apoptosis. Abnormal eutopic endometrium (EUE), which is located in an anatomical position, influences the proliferation of ectopic endometrial tissue (ECE) through the modulation of various molecular pathways. This modulation increases VEGF expression, which in turn promotes angiogenesis in ECE. Additionally, it secretes multiple substances that facilitate ECE growth, differentiation, and resistance to apoptosis [121]. The aberrant expression of proapoptotic genes such as BAX in ectopic lesions, driven by factors such as CXCL8 and ERK signaling, enhances lesion persistence. Epigenetic regulators, such as miR-196b and the lncRNA MALAT1, target PI3K-AKT, cMyc, and estrogen receptor beta (ESR2) signaling to exacerbate resistance to apoptosis.

**Fig. 4:**
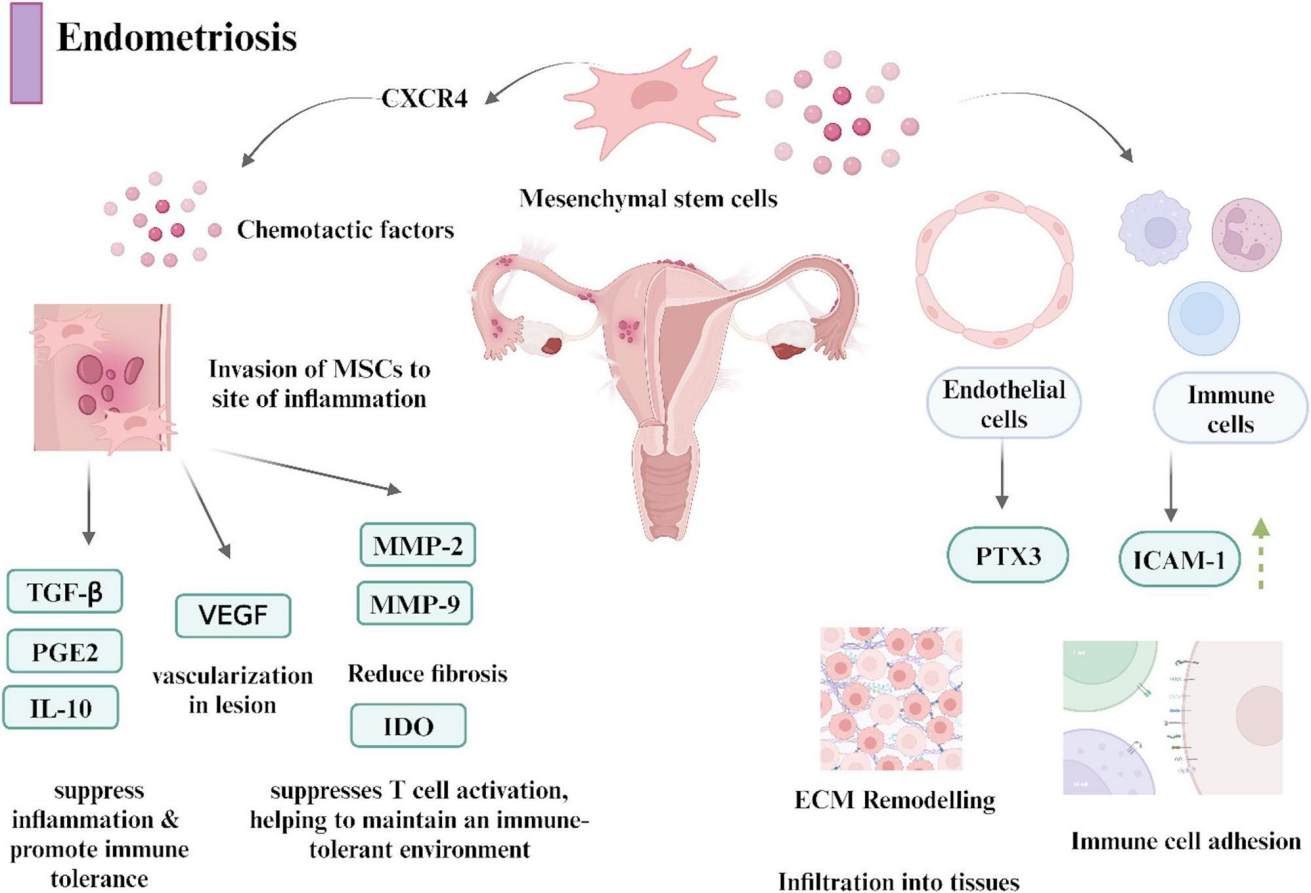
Role of mesenchymal stem cells in managing endometriosis. The MSCs respond to chemotactic factors from the damaged endometriosis alleviation. They suppress the inflammatory markers like TGF-b, PGE2, and IL10), promote vascularization of lesions (VEGF), reduce fibrosis via matrix metalloproteinases (MMP-2 and MMP-9), inhibit the T cell activation (IDO), and facilitate ECM remodeling (PTX3, ICAM, CXCR4). Images were created using Biorender software

Stem cell therapy offers a nuanced therapeutic intervention for endometriosis, moving beyond the traditional methods to target the underlying mechanisms of the disease (Fig. 4). The regenerative potential of transplanted MSCs surpasses that of the simple differentiation processes. MSCs release numerous bioactive molecules that mitigate scarring, modulate inflammatory and immune responses, and activate tissue-specific progenitor cells. MSCs also differentiate into epithelial, stromal, and endothelial cells of the endometrium promoting regeneration and restoring fertility.

**i) Preclinical Evidence**

BM-MSCs can differentiate into endometrial and endothelial cells and potentially regenerate the uterine endometrium, thereby successfully restoring menstruation [122]. In an experimental study, injecting adipose-derived MSCs (2 × 10^6^ cells) into an endometrial rat model successfully reduced the size of the endometrial lesions and the cysts. A significant reduction in chronic inflammatory responses was evident within the endometrium, as evidenced by decreased CD68-positive macrophages and diminished expression of proinflammatory factors TNFα, IL6, and IL1β. Furthermore, the reduced levels of the immunomodulatory cytokines INFγ, TGF-β, and IL-10 observed in these animals further benefit chronic inflammatory conditions [123].

Moreover, injecting the secretome obtained from ADMSCs (1 mL) inhibited the development of endometriosis in a mouse model and improved pregnancy outcomes. The observed effect was correlated with the suppression of intercellular adhesion molecule-1 (ICAM-1) and VEGF in conjunction with increased expression of the endometrial receptivity markers LIF, HOXA10, and HOXA11. PTX3, which functions as a soluble pattern recognition receptor with antiangiogenic, anti-inflammatory, and anti-scarring effects, further contributes to therapeutic potential as it improves the implantation potential of embryos in endometrial tissues. Polarization of macrophages to the M2 subtype can be achieved using HC-HA PTX3 [124].

Exosomes derived from MSCs (MSC-Exos) as cell-free therapeutic agents for treating endometriosis are gaining traction because of their ability to modulate multiple pathways involved in inflammation, apoptosis, and angiogenesis [125]. Exosomes derived from MenSCs significantly reduce the expression of inflammatory markers in endometrial cells, thereby reducing lesion formation in endometrial cells in vitro [126]. MSC-Exos also promote apoptosis in ectopic endometrial cells by downregulating proinflammatory factors and inhibiting angiogenesis via the transfer of miR-16 and miR-150, thereby decreasing the expression of VEGF [127]. They also regulate immune cells by targeting macrophages and T-regulatory cells to restore immunological homeostasis in the peritoneal cavity [128]. Thus, MSCs play a multifaceted role in the treatment of endometriosis by regulating cellular communication and tissue dynamics. The use of exosomes derived from endometrial epithelial cell-derived exosomes for targeted delivery of miRNA to ectopic endometrial tissue to attenuate metastasis in ectopic endometrial tissue is a promising approach for mitigating the endometriosis environment [129]. This method also highlights the potential of exosomes as a means of targeted therapy by acting as a tissue-specific vector for treating endometriosis, thereby enhancing therapeutic efficacy while minimizing off-target effects. The possibility that MSCs influence endometrial pathology by relieving pain and restoring fertility will be crucial for advancing therapeutic options for endometriosis research.

**ii) Clinical Evidence**

Currently, no clinical trials have been registered in the Clinical Trial Registry that investigated the use of stem cells in the treatment of endometriosis. Further preclinical studies are needed to provide robust supporting evidence regarding the efficacy and safety of stem cell-based interventions in endometriosis. Moreover, the transition from preclinical findings to clinical applications requires a thorough understanding of the optimal types of stem cells to used, methods of delivery, and appropriate patient populations that might benefit from such treatments.

#### Thin endometrium

An endometrial thickness exceeding 6–7 mm is critical for developing egg nests and achieving a successful pregnancy [130]. A thin endometrium, characterized by an endometrial thickness of less than 7 mm during the mid-luteal phase, is a critical factor associated with infertility. This condition may impede embryo implantation and result in suboptimal pregnancy outcomes. Implantation in a thin endometrium is associated with an increased risk of miscarriage [130]. Insufficiently developed endometrial lining may fail to provide the requisite blood flow and nutrients essential for maintaining pregnancy, potentially resulting in early pregnancy loss. Hormonal imbalances, inadequate blood circulation, surgical procedures, infections, and prolonged use of hormonal contraceptives or ovulation-inducing agents can adversely affect endometrial thickness [131].

**i) Preclinical Evidence**

Stem cells as a treatment modality were studied by Jing et al. [132] to improve the endometrial thickness in ethanol-induced thin endometrial rat models. Intravenous administration of BM-MSCs improved endometrial thickness by inducing the expression of cytokeratin, vimentin, integrin αγβ3, and leukemia inhibitor factor. These are essential for creating a receptive endometrial environment, particularly during implantation. A notable decrease in the expression of proinflammatory cytokines, such as tumor necrosis factor-α (TNF-α) and interleukin-1β (IL-1β), and significant upregulation of the expression of anti-inflammatory cytokines, such as fibroblast growth factor-β (bFGF) and interleukin-6 mRNA was reported. In addition to their role in immune modulation, they also play essential roles in improving cell adhesion and uterine receptivity. In a similar study, systemic administration of BM-MSCs to an ethanol-induced thin endometrium mouse model increased endometrial thickness and the expression of LIF, IL-1β, and integrin β-3. Decreases in matrix metalloproteinases 2 and 9 involve extracellular matrix degradation and remodeling, which are essential for implantation. These results are supported by the increased pregnancy rates and litter sizes in these rodent models. Yi et al. [133] attributed the therapeutic efficiency of BM-MSCs to the presence of the CXCL12 protein in the secretome. Additionally, increased endometrial thickness was obtained after the transplantation of autologous MenSCs through paracrine activity, as discussed in the previous sections, or through the ability of MenSCs to differentiate into endometrial cells [134]. Chen et al., [135], also conducted a similar study in which the restoration of endometrial thickness, gland numbers and blood vessel formation resulted in an increased pregnancy rate. Orthotopic transplantation of BM-MSCs in rabbits also reported similar results, with a decrease in the area of endometrial fibrosis due to the downregulation of fibrosis markers and interstitial markers, as well as upregulated E-cadherin expression. This study indicated the ability of BM-MSCs combined with estrogen to promote the differentiation of stem cells into endometrial epithelial cells through the synergistic effect of EMT by activating the Wnt/β-catenin signaling pathway [136]. The successful use of collagen scaffolds for stem cell transplantation for improved repair in rat IUA model was reported by Xin et al., [137].

**ii) Clinical Evidence**

Implantation of collagen scaffold loaded with U-MSC (1 × 10^7^ cells) into the uterine cavity resulted in increased endometrial thickness and decreased intrauterine adhesion scores after 3 months of treatment. An increase in Esstrogen receptor α (ERα), vimentin, Ki67, and von Willebrand factor (vWF) and a decrease in ΔNP63 expression level suggested that increased proliferation and neovascularization were observed in endometrial sections. A pregnancy rate of 38% (10/26) was achieved posttreatment, with no apparent congenital disabilities or placental complications [138]. Currently, two successful clinical trials are available in the registry, that have resulted in successful pregnancies after the administration of BM-MSC and U-MSC into the endometrium (Table 4). These studies highlight the promising role of stem cell transplantation in addressing endometrial dysfunction and improving reproductive outcomes.

#### Asherman syndrome (AS)

Repeated curettage procedures and endometriosis destroy the uterine cavity, leading to intrauterine adhesions and the absence of a functional endometrium in multiple areas. Women diagnosed with AS often have atrophic endometria measuring ≤ 4 mm, leading to fertility challenges, menstrual irregularities, and recurrent pregnancy loss [139]. Several studies have indicated that administration of BM-MSCs improves endometrial regeneration in a murine model of AS [139–142]. Endometrium restoration in patients with Asherman syndrome was correlated with improved reproductive outcomes.

**i) Preclinical Evidence**

BM-MSCs improved immunomodulatory activity and enhanced differentiation potential. The implantation of silk fibroin/polycaprolactone electrospun nanofibers scaffolds laid with ADMSCs-SF/PCL (2.5 × 0.5 cm^2^, 1 × 10^6^ ADMSCs) into the uterine cavity of the IUA rat model. This implant successfully reversed endometrial damage with the regeneration of the glands, angiogenesis, and reversal of fibrosis. The immune microenvironment was remodelled in the uterine cavity with an increased infiltration of NK cells and a normalised Th1/Th2 bias [143]. In a similar study, the transplantation of human bone marrow-derived clonal MSCs (cMSCs), heterogenous parental MSCs (hMSCs), or cMSCs-derived EV subpopulations (EV20K to a mechanically induced AS model resulted in endometrial repair and restored reproductive functions in all the treated groups. cMSCs migrated to the uterus and downregulated proinflammatory factor TNFα and upregulated anti-inflammatory cytokine IL-10, resulting in increased implantation rate by improving endometrial receptivity cytokines VEGF and LIF [144].

The use of collagen scaffolds loaded with BMMSCs implanted near damaged endometrial tissues resulted in the secretion of specific growth factors, such as thrombospondin-1 and insulin-like growth factor-1. This inhibited fibrosis, promoted angiogenesis and enhanced endometrial receptivity in AS animal models [145].

**ii) Clinical Evidence**

MenSC transplanted into the uterus of patients with AS showed a significant improvement of the endometrial thickness resulting in a pregnancy rate of 41.7% (5 out of 12) as reported by Ma et al., [146]. Men SC isolated from infertile patients also effectively reduced endometrial damage promoting angiogenesis and reducing inflammation [147]. Studies have demonstrated that UC-MSCs can differentiate into endometrial cells, potentially assisting in repairing damaged endometria and improving fertility [148]. However, despite multiple efforts, peripheral blood stem cell transplantation (PBSCT) has failed to achieve successful engraftment of donor stem cells in the recipient uterus, as observed in both a macaque model and human subjects [149]. Currently, research is focused on clinical trials promoting endometrial regeneration and reducing damage to human endometrial stromal cells. These findings have significant implications for development of new therapeutic strategies aimed at treating endometrial disorders, highlighting the essential contribution of stem cell research in reproductive medicine.

Although the use of stem cells as therapeutic agents, even though it shows promising results, the major limitations include the absence of long-term safety and efficacy data. A major concern is the tumorigenic risk of stem cells, particularly when pluripotent stem cells are used for treatments. Additionally, the use of autologous stem cell transplantion has not been discussed in any of these clinical trials. Ultimately, for clinical translation and widespread adoption of these technologies, the scientific community must address the critical areas.

## Good manufacturing practices for stem cell therapies in reproductive disorders

With the advancement of therapeutic approaches toward clinical translation, adherence to stringent Good Manufacturing Practices (GMP) is mandatory to ensure the safety, purity, potency, and consistency of the stem cell products. This maximises their therapeutic efficacy while minimising risks to patients. Thus the development and validation of comprehensive GMP-compliant quality control assays are fundamental for transforming the landscape of female reproductive healthcare. The key practices include maintaining xeno-free culture conditions, rigorous quality control, and standardised protocols throughout the manufacturing process.

### Xeno-Free culture conditions

To minimise the immunogenicity and ensure patient safety, regulatory guidelines encourage the culture of stem cells in xeno-free environments. This approach ensures enhanced compatibility of stem cells for therapeutic applications, especially in reproductive health [150]. When culturing stem cells in vitro, it is essential to use certified reagents and equipment to minimise contamination risks. This standardised approach not only fosters a safer clinical environment but also aligns with the growing emphasis on ensuring the safety and efficacy of cell-based therapies [151].

### Quality control and testing

Continuous monitoring of manufacturing processes, such as cell culture and expansion, is necessary to ensure that products meet predetermined specifications [152]. Quality control testing includes testing for sterility, potency, and genetic stability of stem cell lines to ensure the safety of stem cell products [153]. For a consistent supply of high-quality stem cells for clinical use, master cell banks (MCBs) under cGMP conditions have been established [154]. Along with production and quality control, stringent release criteria must be established for stem cell products, including various assays for sterility, viability and genomic stability [155]. Sterility assays include assessment for microbial load (bacteria, fungi and yeast) in the final product, Mycoplasma testing by PCR or ELISA base techniques and endotoxin testing by the Limulus Amoebocyte Lysate (LAL) assay. Flow cytometric analysis for cell viability, cell count, identity testing and immunophenotyping was carried out. Short Tandem Repeat Analysis was used to confirm the genetic identity of the cell line and rule out cross contamination. Various purity assays are recommended, such as immunostaining and residual impurity check for serum, cytokines and other viral particles [155].

Potency assays confirm the critical functional test, which measures the biological activity and intended therapeutic effect of stem cells [156]. These assays are product-specific and aligned with the stem cell mechanism of action. General assays include in vitro differentiation and cell-based functional assays that measure the secretion of growth factors, immunomodulatory effects and other biological activities. Assessment for chromosomal abnormalities after long-term culture by karyotyping analysis, both in the master cell bank and at the end of production, is recommended [157]. Other methods, such as array comparative genomic hybridisation and qPCR, can also be used to detect small changes.

### Standardized manufacturing protocols

Standardised operating protocols (SOPs) for cell reprogramming, expansion, and storage must adhere to cGMP standards to ensure reproducibility and reliability of the therapeutic product [158]. The use of specific reagents and equipment compliant with cGMP is critical for maintaining the integrity of stem cell products [159]. The implementation of SOPs is crucial for documenting all processes ensuring traceability and facilitating regulatory compliance for product manufacturing and release.

Implementing these cGMP guidelines not only enhances the reliability of stem cell products but also addresses the challenges faced by academic institutions in establishing compliant manufacturing facilities [160]. Overall, adherence to cGMP and associated quality control measures is critical for advancing stem cell therapeutics and facilitating regulatory approval. While the focus on cGMP practices is crucial for the advancement of stem cell therapies, challenges remain in harmonising regulations across jurisdictions and addressing ethical concerns related to donor tissue use.

## Ethical considerations and regulatory challenges

Stem cell research holds immense promise in the management of female reproductive disorders. However, it is fraught with ethical and regulatory complexities that must be carefully navigated before such therapies can be widely adopted. One of the most pressing moral concerns in clinical trials is ensuring informed consent. Patients facing reproductive challenges can be vulnerable and eager to try novel therapies, which raises the risk of undue influences, including therapeutic misconceptions. Therefore, clear, transparent communication about potential benefits, limitations, and risks is vital to protect patient autonomy and well-being [161, 162].

In addition to consent, equity in access represents a critical ethical issue. Advanced stem cell therapies often require specialized equipment and facilities for harvesting, manipulation, and transplantation [163]. The high cost and logistical demands risk of these procedures make them inaccessible to patients in low-resource settings, potentially exacerbating healthcare disparities. Moreover, the commercialization of unproven stem cell treatments, which are frequently advertised directly to patients underscores the danger of exploitation. Unscrupulous clinics may skirt strict regulatory oversight, offering therapies without sufficient safety or efficacy data [161]. These practices endanger vulnerable patients and undermine ethically conducted research.

From a governance standpoint, oversight and transparency are essential for minimizing unethical practices. Guidelines such as those proposed by the International Society for Stem Cell Research (ISSCR) emphasize rigorous preclinical validation, transparent reporting of results, and approval by independent ethics committees or institutional review boards [161]. These measures ensure that innovative therapies are developed and tested responsibly, thus balancing scientific progress with patient safety and rights.

### Regulatory challenges: A global perspective

Regulatory agencies, including the U.S. Food and Drug Administration (FDA) and the European Medicines Agency (EMA), face hurdles in establishing consistent criteria for approving novel stem cell-based products. Classifications of stem cell products as “drugs,” “tissues,” or “advanced therapy medicinal products” often dictate approval pathways, manufacturing requirements, and post-market surveillance [162, 164]. This variability can create “safe havens” and confusion, hindering international collaboration. Moreover, the long-term safety and efficacy profiles of stem cell therapies for reproductive disorders remain uncertain, making robust clinical trials and comprehensive follow-up essential [163].

### Regulatory challenges in the Indian context

In India, the Central Drugs Standard Control Organization (CDSCO) serves as the national regulatory authority for pharmaceuticals and medical devices, including advanced therapy medicinal products (ATMPs). The Indian Council of Medical Research (ICMR), alongside the Department of Biotechnology (DBT), has formulated the *National Guidelines for Stem Cell Research (NGSCR*,* 2017)*, which underscores the ethical and scientific imperatives for both basic and translational research [165]. These guidelines mandate that stem cell-based interventions adhere to rigorous standards for informed consent, safe cell manipulation, and transparent reporting of data. However, the pathway to regulatory approval can be complex, partly owing to ambiguities in categorizing cell products as “minimally manipulated” or “substantially manipulated.” Such distinctions affect the applicable approval processes, manufacturing requirements, and monitoring protocols. Despite this regulatory framework, the compliance remains inconsistent. Several private clinics operate with limited oversight and offer treatments that are unsupported by robust clinical data. This underlines the need for more stringent enforcement, harmonized standards, and increased collaboration among government agencies, research institutions, and healthcare providers.

In summary, while stem cell treatments exhibit tremendous therapeutic potential for female reproductive disorders, ethical and regulatory considerations are paramount to ensure responsible research, patient safety, and equitable access. Upholding rigorous guidelines, bolstering international collaboration for consistent standards, and guarding against unproven therapies are essential steps toward safely harnessing the full promise of these novel interventions.

## Future perspectives

The future of stem cell therapy presents significant promise in addressing female reproductive disorders, particularly with continuous advances in scaffolding and biotechnology. With the revolution in three-dimensional scaffolding methods, stem cell transplantation has evolved. Incorporating a biomimetic scaffold designed by adding an extracellular matrix and biocompatible materials facilitates the incorporation of growth factors and signaling molecules, resulting in enhanced cellular and tissue integration. This can improve the transplantation of stem cells into the target tissue, thereby improving the reproductive outcome. Future research efforts to develop nanostructured scaffolds can enable precise cellular guidance over controlled release mechanisms and surface modification, thereby improving cellular adhesion. These advancements are crucial for optimizing the microenvironment of transplanted stem cells, which can significantly influence their functionality and host tissue integration.

As biotechnology progresses, comprehensive genetic screening and mapping of epigenetic modifications in reproductive disorders are being actively performed. This approach will help identify patient-specific molecular markers and customized stem cell intervention strategies to meet individual needs. The generation of iPSCs through direct cellular reprogramming from patient-specific cell lines that can closely align with patients’ genetic profiles represents a new hope for patients facing infertility challenges. The translation of ongoing clinical trials is expected to provide evidence-based validations for these technologies. These studies are instrumental in refining treatment protocols and establishing standardized clinical care practices for stem cell therapies. These findings enhance our understanding of the efficacy and safety of stem cell interventions and improving patient outcomes.

## Conclusion

Stem cell research on female reproductive disorders has progressed significantly over the past decade, reflecting both the increasing sophistication of laboratory techniques and a more nuanced understanding of the reproductive pathophysiology. This review highlights the considerable therapeutic potential of various stem cell populations, such as iPSCs and MSCs, in conditions ranging from ovarian insufficiency to endometrial dysfunction. Notably, preclinical investigations have yielded promising results, revealing that stem cells can potentially restore or enhance uterine and ovarian function through mechanisms involving tissue regeneration, anti-inflammatory effects, and the modulation of local immune responses. These findings, albeit preliminary in human contexts, underscore the exciting prospects for stem cell-based therapies in reproductive medicine.

Despite these advances, several critical challenges remain before stem cell therapies can be adopted in clinical practice. The first challenge lies in achieving consistent and reproducible clinical outcomes. Variations in stem cell sources, culture protocols, and transplantation techniques can lead to heterogeneous therapeutic efficacy and safety profiles. Moreover, as human trials progress, robust cell characterization and standardization methods are imperative. Without these factors, interpreting outcomes across different studies becomes problematic, impeding the development of evidence-based treatment guidelines. Ethical and regulatory considerations also loom large: ensuring patient safety requires long-term monitoring for potential adverse events such as tumorigenicity or ectopic tissue formation. Ethical concerns regarding sourcing and manipulating cells to achieve therapeutic endpoints must be addressed cautiously and transparently.

Furthermore, although considerable research has focused on the mechanistic action of stem cells in regenerative processes, gaps remain in our understanding of how these cells interact with the patient’s immune system, the hormonal milieu, and the underlying pathology. Bridging these knowledge gaps will necessitate interdisciplinary collaborations integrating reproductive biology, regenerative medicine, immunology, and bioengineering expertise. In parallel, large-scale, well-designed clinical trials are essential to generate high-quality evidence on safety, efficacy, and optimal protocols. Establishing standardized guideline, cell type selection, dosing strategies, and transplantation procedures will enable researchers and clinicians to build consistent, comparable data sets.

In conclusion, stem cell therapies represent a compelling new frontier for the treatment of female reproductive disorders. With meticulous research design, ethical oversight, and informed clinical translation, the promise demonstrated in preclinical models can be harnessed to deliver safe, and effective regenerative treatments. This approach will pave the way for improved patient outcomes and potentially transformative changes in the reproductive healthcare landscape. Ultimately, the successful clinical translation of these therapies will not hinge on a single breakthrough, but on a concerted, interdisciplinary effort. We call for the establishment of international consortia to standardise protocols, share negative data transparently, and harmonise regulatory approvals. Only then can we responsibly bridge the gap between preclinical promise and tangible patient benefits in reproductive medicine.

## Data Availability

No datasets were generated or analysed during the current study.
